# Intracranial extravasation of contrast medium during diagnostic CT angiography in the initial evaluation of subarachnoid hemorrhage: report of 16 cases and review of the literature

**DOI:** 10.1186/2193-1801-2-413

**Published:** 2013-08-28

**Authors:** Hitoshi Kobata, Akira Sugie, Erina Yoritsune, Tomo Miyata, Taichiro Toho

**Affiliations:** Osaka Mishima Emergency Critical Care Center, 11-1 Minamiakutagawacho, Takatsuki, Osaka, 569-1124 Japan

## Abstract

**Introduction:**

Three-dimensional CT angiography (3D-CTA) is increasingly used in the initial evaluation of subarachnoid hemorrhage (SAH). However, there is a risk of aneurysm re-rupture in the hyperacute phase. We sought to clarify the incidence of re-rupture and characterize the subgroup in which extravasation of contrast media was seen on 3D-CTA.

**Methods:**

We examined the records of 356 consecutive patients presenting to our institution with non-traumatic SAH between October 2003 and December 2011. After resuscitation, patients with poor grade SAH underwent CT then 3D-CTA while sedated, mechanically ventilated and with a target systolic blood pressure of 120 mmHg.

**Results:**

336 patients underwent 3D-CTA; 20 died without return of spontaneous circulation. Extravasated contrast medium was seen in 16 (4.8%), 15 (4.5%) at the initial evaluation. Their World Federation of Neurosurgical Societies Grade was V; one patient was resuscitated from cardiac arrest. The mean times from onset to arrival and to CTA were 43.7 minutes and 71.8 minutes, respectively. Ten patients (62.5%) had episodes suggestive of aneurysm re-rupture before 3D-CTA. Surgical clipping, evacuation of hematoma and wide decompressive craniectomy was completed in six patients and one underwent coil embolization. Two of 16 patients survived: one with moderate disability and one made a good recovery.

**Conclusions:**

Contrast extravasation was detected by 3D-CTA in 4.5% of cases despite intensive resuscitation, suggesting that continuous or intermittent rebleeding may occur frequently in the hyperacute phase. The consequences of rebleeding are devastating; however, favorable results can be obtained with immediate aneurysm repair with decompression and intensive neurocritical care.

**Electronic supplementary material:**

The online version of this article (doi:10.1186/2193-1801-2-413) contains supplementary material, which is available to authorized users.

## Introduction

Owing to advances in imaging technology, three-dimensional computed tomography angiography (3D-CTA) is now widely used as the first-line diagnostic modality to identify ruptured aneurysms in patients with subarachnoid hemorrhage (SAH) (Connolly et al. 2012; Diringer et al. 2011). CTA can easily be performed after SAH has been diagnosed using unenhanced CT in an emergency, allowing aneurysms to be secured earlier and by less invasive means. It is also recognized that aneurysmal re-rupture is less likely to occur with CTA compared with conventional digital subtraction angiography (DSA) (Tanno et al. 2007). However, it is increasingly evident that the risk of re-rupture is not negligible, especially in the hyperacute phase (Tsuang et al. 2012; Suzuki et al. 2012); there is an increasing number of reports of aneurysmal re-rupture during CTA (Nakatsuka et al. 2002; Gosselin & Vieco 1997; Nakada et al. 2000; Holodny et al. 2003; Josephson et al. 2004; Ryu et al. 2005; Pérez-Núñez et al. 2006; Hashiguchi et al. 2007; Im et al. 2007; Nagai et al. 2008; Desai et al. 2009; Sholtes et al. 2011), the vast majority resulting in a devastating outcome for the patient regardless of subsequent treatment (Tsuang et al. 2012). Because rebleeding is the most crucial preventable cause of death in patients hospitalized after SAH (Broderick et al. 1994), it is of paramount significance to avoid rebleeding during the initial resuscitative and diagnostic procedure. Early and intensive cardiopulmonary and neurological support should be encouraged (Komotar et al. 2009).

The aims of this study were to examine the incidence of contrast medium extravasation in patients with SAH undergoing CTA and describe the consequences for the patients. We discuss the implications for the immediate management of contrast medium extravasation, and initial resuscitation, especially for patients in the hyperacute phase with poor grade SAH (Grades IV and V according to the World Federation of Neurosurgical Societies (WFNS) criteria (Drake 1988)) and cardiac arrest (CA).

## Methods

### Study design

We retrospectively reviewed the records of 356 consecutive patients with non-traumatic SAH transferred to our hospital between October 2003 (when 16-detector row CT became available in our institution) and December 2011. This study was performed in accordance with the guidance of the Helsinki Declaration and all patients provided written informed consent before the publication of this report.

### Episodes suggesting re-rupture

We defined re-rupture as a sudden deterioration in conscious level witnessed by paramedics, hospital staff or bystanders following the initial ictus, or the occurrence of a generalized epileptiform seizure after the initial onset of SAH. For patients referred from other hospitals with a diagnosis of SAH made by CT, we defined re-rupture as an increase in the volume of intracerebral hemorrhage as assessed on arrival CT. We excluded subtle “warning” sings such as mild headache, faintness, or nausea from SAH onset.

### Initial resuscitation protocol

Following prompt evaluation of vital signs and neurological status immediately after arrival, patients with poor grade SAH were carefully resuscitated with the aim of maintaining the systolic blood pressure (BP) at 120 mmHg, sedated, intubated and mechanically ventilated, before undergoing CT and CTA (Kobata et al. 2007). Sedation was achieved by means of buprenorphine 0.4 mg (5–8 μg/kg) and midazolam 10 mg (0.125–0.2 mg/kg). Endotracheal intubation was undertaken after the administration of vecuronium 10 mg (0.125–0.2 mg/kg), with care taken to avoid hypertension. Invasive monitoring, including a radial artery catheter, was established. Propofol 100–500 mg (1.25–10 mg/kg) was titrated for sedation and in order to control BP. If necessary, nicardipine 2–10 mg (25–200 μg/kg) was also given (Kobata et al. 2007). Aminocaproic acid or tranexamic acid was not administered.

For patients who arrived in CA, cardiopulmonary resuscitation was administered and brain CT was performed to identify the cause. CTA was performed in 38 patients with return of spontaneous cardiac circulation (ROSC) who remained hemodynamically stable. Twenty patients without ROSC did not qualify for immediate CTA.

### CT and CTA imaging protocol

Immediately after resuscitation all patients underwent brain CT, the results of which were classified using the Modified Fisher classification (Frontera et al. 2006). During CT and CTA, deep sedation and mechanical ventilation were maintained with continuous BP monitoring.

All CTA was performed with a 16-detector multislice Aquilion CT scanner (Toshiba, Inc., Tokyo, Japan) with the following parameters: 120 kV/300 mA; 11 helical pitch; 0.5 mm slice thickness; 0.3 mm reconstruction pitch and 0.75 seconds/r gantry rotation time. For enhancement, a 100 ml of Omnipaque 350 (Daiichi Pharmaceutical Co., Tokyo, Japan) nonionic contrast medium was delivered into the antecubital vein at a rate of 3 ml/s by means of a power injector. For optimal intracranial contrast enhancement, the delay between the start of injection and the start of scanning was determined for each patient using a bolus-tracking technique, mostly 20–25 s. Scanning range was from the first cervical vertebra to a point 1 cm above the lateral ventricles. The data obtained were transferred to a workstation (Aquarius NetStation version 1.4.3.0, TeraRicon Inc., San Mateo, California) for further processing to reconstruct two- and three-dimensional images viewed from optional different directions. Two board-certified neurosurgeons (H. K. and A. S.) and radiological technologists operated the workstation and interpreted the 2D- and 3D-CTA images as well as the source image.

### Aneurysm-securing treatment

For patients with evidence of extravasation from the aneurysm, surgical clipping and wide decompressive craniectomy was undertaken as soon as feasible, with the exception of patients with bilateral fixed and dilated pupils after resuscitation, the very elderly over 80 years or those with substantial co-existing systemic disease. During the procedure as much of the hematoma and subarachnoid clot were evacuated as possible, as was extravasated contrast medium. Endovascular coil embolization was chosen if direct surgery was thought disadvantageous, for example basilar tip aneurysm, multiple aneurysms and aneurysms that had regrown after previous clipping.

### Statistical analysis

Statistical analysis of categorical variables was undertaken using the chi-squared test (JMP statistical software version 9.0.0; SAS Institute, Inc., Cary, North Carolina).

## Results

**Figure 1:**
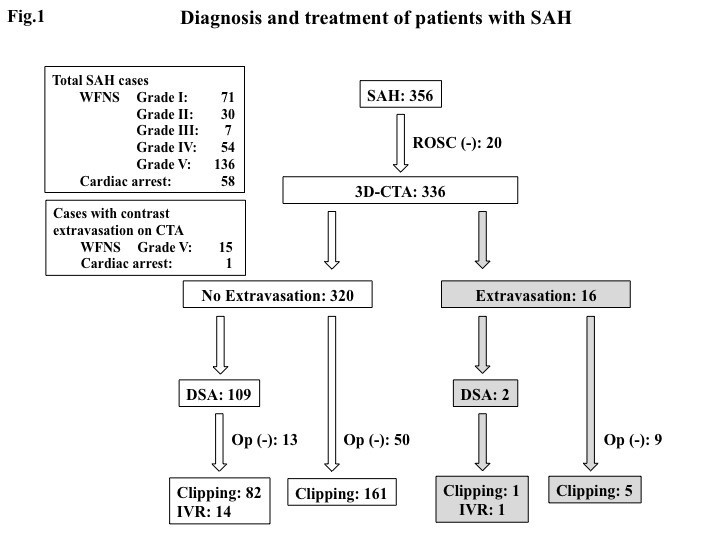
**Flow chart showing diagnostic and therapeutic procedures used in 356 cases of non-traumatic subarachnoid hemorrhage (SAH).** Abbreviations: WFNS, World federation of neurosurgical societies; ROSC, Return of spontaneous circulation; 3D-CTA, 3 dimensional-computed tomography angiography; DSA, Digital subtraction angiography; IVR, Interventional radiology.

The means of diagnosis and treatment of the patients are shown in Figure 1. During the study period, 356 SAH patients were admitted to our hospital, of whom 248 (69.7%) were assessed as poor grade or CA (Grade IV: 54, Grade V: 136, CA: 58). Of these, 336 patients underwent 3D-CTA, including 38 patients who arrived in CA and in whom ROSC was achieved. Extravasation was detected on 16 occasions (4.8%), 15 of these (4.5%) during the initial evaluation following resuscitation.

The demographic data of the 16 patients with contrast medium extravasation are shown in Table 1. In 15 patients, the WFNS grade was assessed to be V; the remaining patient had been successfully resuscitated after CA. The presence of pulmonary edema and stunned myocardium was often documented. The most frequent location of ruptured aneurysms was the anterior communicating artery. In 11 patients in whom the ictus had been witnessed, the mean time from onset to arrival and to CTA was 43.7 minutes and 71.8 minutes, respectively. Of note, 10 (62.5%) had clinical episodes suggesting aneurysm re-rupture before 3D-CTA was undertaken, compared with 18.4% (59/320) of patients in whom there was no evidence of contrast medium extravasation (p <0.001, chi-square test). The mean systolic BP of the group of patients who subsequently experienced extravasation was 164 mmHg on arrival, which was reduced to 120 mmHg just before CTA and then remained stable during CTA. Fourteen patients died in the acute stage whereas two recovered well after emergent surgical intervention and intensive neurocritical care: one made a good recovery and one recovered with moderate disability.

**Table 1:** **Demographic data of 16 patients with contrast medium extravasation on computed tomography angiography (CTA)**

Characteristic	Value
Sex (M/F)	5/11
Age (mean ± SD, range)	71.6 ± 10.6, 53 - 96
WFNS Grade	V: 16 (incl, 1 resuscitated from CA)
Aneurysm location	
AcoA	6
ICA	4
MCA	3
VA	2
ACA	1
Cardiopulmonary complications	
Pulmonary edema	5
Stunned myocardium	4
Antithrombotic agents	aspirin: 2
	aspirin and warfarin: 1
Episode of rerupture before CTA	10
Time from onset to arrival (minutes)*	43.7 ± 27.5
Time from onset to CTA (minutes)*	71.8 ± 25.4
Systolic BP (mmHg)	
On arrival	163.8 ± 62.0
Before CTA	119.5 ± 41.2
After CTA	118.7 ± 44.1
Outcome (Glasgow Outcome Scale)	
GR	1
MD	1
D	14

*WFNS* World Federation of Neurosurgical Societies, *CA* cardiac arrest, *CTA* computed tomography angiography, *AcoA* anterior communicating artery, *ICA* internal carotid artery, *MCA* middle cerebral artery, *VA* vertebral artery, *ACA* anterior cerebral artery, *BP* blood pressure, *GR* good recovery, *MD* moderate disability, *D* death.

* Calculated from the 11 patients in whom exact onset time was identified.

**Figure 2:**
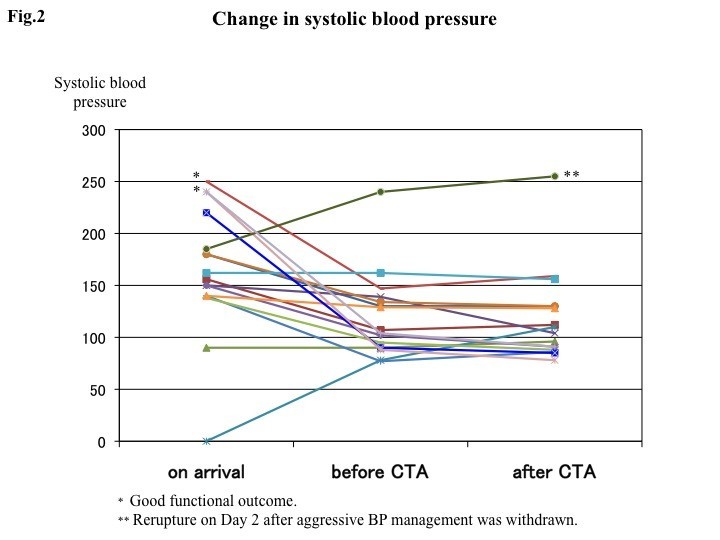
**Graph showing systolic blood pressure on arrival, before 3D-computed tomography angiography (3D-CTA) and after 3D-CTA.** * Good functional outcome. ** Re-rupture on Day 2 after intensive blood pressure management was withdrawn.

Figure 2 shows the systolic BP on arrival, and subsequent changes before and after CTA. Systolic BP on arrival exceeded 160 mmHg in eight patients, which responded to treatment in six patients; however, in two patients BP remained elevated between 150 and 160 mmHg despite treatment. In the two patients with favorable outcomes, initial very high systolic BPs (250 and 240 mmHg) were rapidly controlled before CTA and maintained stable thereafter at 147 and 104 mmHg, respectively. No ischemic lesion was detected on postoperative magnetic resonance imaging studies.

Table 2 details the demographic, clinical and outcome data of each case. All had diffuse dense SAH on CT except for one patient, in whom intraventricular and subdural hematomas were dominant. An emergency surgical clipping following wide decompressive craniectomy was attempted in seven patients, and completed in six. Premature rupture occurred shortly after dural incision on three occasions; clipping was completed in two cases but in the other case an external ventricular drain was placed as severe brain swelling prevented access to the aneurysm. Coil embolization was chosen in one patient in whom 3 aneurysms were detected; one was a de novo aneurysm showing contrast medium extravasation and the others were recurrent aneurysms after previous clipping surgery. Extravasation was also observed from the aneurysm with CTA extravasation during diagnostic angiography.

**Table 2:** **Subarachnoid hemorrhage (SAH) cases in which extravasation was identified during computed tomography angiography (CTA)**

Case	Age	Sex	WFNS Grade	Pupils (mm)	Modified fisher grade	Location	Aneurysm size (mm)	Aneurysm shape	Time form onset to arrival (min)	Time from onset to CTA (min)	Episode of re-rupture before CTA	Blood pressure			Treatment	GOS
												On arrival	Before CTA	After CTA		
1	78	M	5	8.0/7.0	2	l-AC distal	19.5	berry	35-60	55-80	yes	180/90	130/70	130/60	ND	D
2	76	F	5	3.0/3.0	4	Acom	10.4	berry	45-495	84-495	yes	156/90	107/79	112/70	Clipping*	D
3	74	F	5	4.0/4.0	3	r-MCA	2.8	berry	26	66	yes	90/-	90/52	96/47	Clipping	D
4	77	F	5	4.5/4.5	3	l-ICPC	7.8	berry	26	56	no	150/90	139/75	104/81	Clipping	D
5	63	F	CA	6.5/6.5	3	l-MCA	3.4	berry	105	132	yes	0/0	78/48	110/68	ND	D
6	56	F	5	4.0/4.0	3	Acom	7.5	berry	43-133	70-160	no	180/80	134/80	130/80	ND	D
7	77	F	5	ND/1.5	4	r-ICPC	9.7	mult-lob	30	63	yes	140/80	77/54	86/52	GDC*	D
8	66	F	5	3.0/3.0	3	r-ICPC	11.4	mult-lob	28	60	yes	250/170	147/86	159/69	Clipping	MD
9	62	F	5	5.5/5.5	3	Acom	5.5	mult-lob	31	57	no	138/100	95/64	88/60	ND	D
10	53	M	5	6.0/6.0	3	Acom	2.6	berry	80	100	yes	150/110	102/57	91/48	ND	D
11	96	F	5	2.0/2.5	4	Acom	2.5	berry	33-180	48-195	no	162/72	162/72	156/74	ND	D
12	69	M	5	6.0/3.0	3	r-MCA	4.9	berry	66-360	88-382	yes	140/80	129/96	128/74	Clipping*	D
13	74	M	5	6.0/2.0	3	l-IC dissec s/o	ND	dissection	32	61	no	220/110	90/45	85/45	ND	D
14	69	F	5	3.5/3.5	3	Acom	7.9	berry	25	45	no	240/120	88/42	78/40	EVD,ED*	D
15	84	M	5	2.0/2.0	4	l-VA trunk	5.6	fusiform	68	90	yes	185/115	240/84	255/90	ND	D
16	71	F	5	1.5/1.5	3	l-VAPICA	5.9	berry	30	60	yes	240/160	104/74	91/63	Clipping	GR

*WFNS* World Federation of Neurosurgical Societties, *GOS* Glasgow Outcome Scale, *M* male, *F* female, *CA* cardiac arrest, *l* left, *r* right, *AC* anterior cerebral artery, *AcoA* anterior communicating artery, *MCA* middle cerebral artery, *ICPC* internal carotid-posterior communicating artery, *dissec* dissection, *VA* vertebral artery, *VAPICA* vertebral artery-posterior inferior cerebellar artery, *mult-lob* multiple lobules, *EVD* external ventricular drainage, *ED* external decompression, *GR* good recovery, *MD* moderate disability, *D* death. * Rerupture occurred during the surgical or endovascular procedure.

### Illustrated cases

#### Case 12

**Figure 3:**
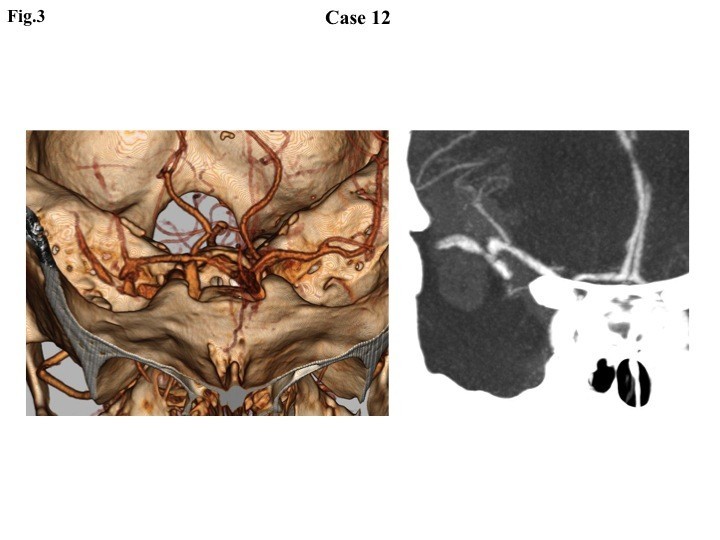
**Three- and two-dimensional computed tomography angiography showing a right middle cerebral artery aneurysm and extravasation of contrast media into the Sylvian hematoma.**

This 69-year-old man was comatose at home and brought to the hospital by ambulance. The estimated time from onset to arrival was between 66 and 360 minutes. His Glasgow Coma Scale (GCS) was 4 (eyes 1, verbal 1, motor 2 [E1, V1, M2]) showing anisocoria with bilateral sluggish reaction to light. Blood pressure was 150/110 mmHg, which was lowered to 129/96 mmHg before intubation. Brain CT after initial resuscitation revealed diffuse thick SAH associated with a large right Sylvian hematoma. CTA performed 22 minutes after arrival revealed an aneurysm at the right middle cerebral artery associated with contrast medium extravasating into the hematoma cavity (Figure 3). He was transferred to the operating room 48 minutes after arrival for surgical clipping and decompression. When the dura mater was opened, the brain herniated through the incision and extravasated blood was expelled. The aneurysm was clipped but the patient did not regain consciousness and died 13 days later.

#### Case 16

**Figure 4:**
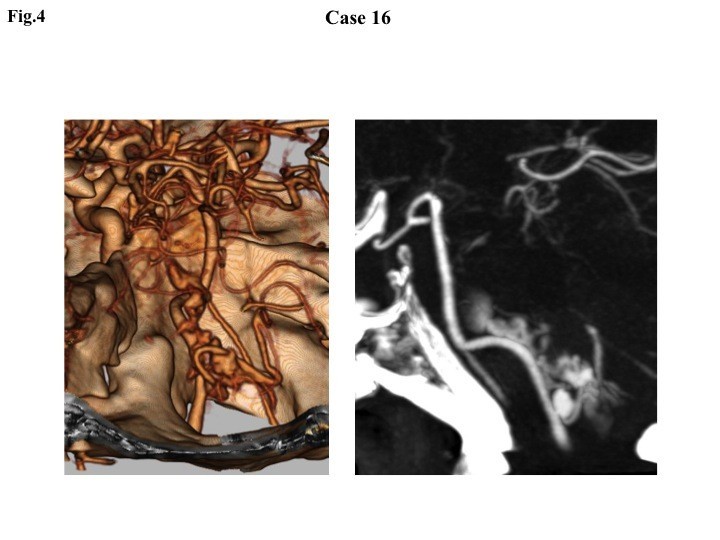
**Three- and two-dimensional computed tomography angiography showing an aneurysm at the left vertebral artery-posterior inferior cerebellar artery junction associated with cauliflower-like extravasated contrast medium.** Lack of drainage indicates that this lesion is not an arteriovenous malformation but a ruptured aneurysm.

This 71-year-old woman was brought to the hospital immediately after a witnessed collapse at midnight. On arrival 30 minutes later, she had miotic non-reactive pupils and a GCS score of 3. Blood pressure was 240/160 mmHg. Brain CT and CTA were undertaken 30 minutes after arrival and resuscitation. Brain CT showed dense diffuse SAH predominantly around the medulla and pons; CTA revealed a cauliflower-like high-density lesion around the left vertebral artery (Figure 4). The residents on duty interpreted the latter finding as an arteriovenous malformation. She was kept sedated and underwent DSA 11 hours after onset, in which an aneurysm at the junction between the vertebral artery and posterior inferior cerebellar artery (VA-PICA) was confirmed. Surgical clipping was completed by means of a wide suboccipital craniectomy 14 hours after onset. No aneurysm re-rupture was encountered during DSA or surgery. She recovered well and was discharged to rehabilitation institute. After a ventriculoperitoneal shunt operation for hydrocephalus, she became independent. Outcome at 6 months was assessed as a good recovery by Glasgow Outcome Scale (Jennett & Bond 1975) and 1 by the modified Rankin Scale (van Swieten et al. 1988).

## Discussion

### Episode of re-rupture

Initial and recurrent bleeding are the major causes of death following SAH. Many patients with SAH die or deteriorate before arrival at hospital or shortly afterwards. Episodes of rebleeding have been observed in the ambulance in 12.9% and shortly after admission in 6.7% (Kobata et al. 2007) of cases, or in the ambulance or at the referring hospital in 13.6% (Ohkuma et al. 2001) of cases. These moribund patients may no longer be candidates for aneurysm repair. Rebleeding is most common within a few hours after onset (Kobata et al. 2007; Ohkuma et al. 2001). Because rebleeding is thought to be potentially avoidable, initial resuscitation to mitigate rebleeding risk and cardiopulmonary stabilization are crucial elements of the treatment of SAH. CTA is considered less likely to trigger re-rupture compared with conventional DSA (Tanno et al. 2007) and is straightforward to perform immediately after confirmation of SAH by CT (Tsuang et al. 2012). However, there are an increasing number of reports of rebleeding, manifest by extravasation of contrast medium, during CTA (Tsuang et al. 2012; Suzuki et al. 2012; Nakatsuka et al. 2002; Gosselin & Vieco 1997; Nakada et al. 2000; Holodny et al. 2003; Josephson et al. 2004; Ryu et al. 2005; Pérez-Núñez et al. 2006; Hashiguchi et al. 2007; Im et al. 2007; Nagai et al. 2008; Desai et al. 2009; Sholtes et al. 2011).

### Incidence of extravasation during CTA

Several studies have reported a surprisingly high incidence of extravasation during CTA, ranging between 14.5% (nine out of 62 cases) (Tsuang et al. 2012) and 17.9% (five out of 28 cases, including two possible cases) (Nakatsuka et al. 2002). Moreover, when more sensitive techniques such as multiphase dynamic-enhanced CT (4D-CTA) are used, the incidence may be as high as 25.5% (13 out of 51 cases) or even 42.3% (11 out of 26 cases within 2 hours of onset) (Suzuki et al. 2012). With intensive resuscitation and control of hypertension, extravasation was seen on CTA in 4.5% of cases in our series. Our findings confirm that the majority of patients showing extravasation on CTA were those with poor grade SAH examined in the hyperacute phase (Connolly et al. 2012). In our series, 10 out of 16 patients with extravasation on CTA had experienced episodes of re-rupture before CTA. The occurrence of early, continuous or frequent intermittent re-rupture may be part of the natural course of poor grade SAH.

### Imaging features of extravasation

Extravasation on 3D-CTA has variously been described as: cap-shaped (Nakatsuka et al. 2002); corkscrew-like (Nakatsuka et al. 2002); shaped like a twisted ribbon (Ryu et al. 2005); conical (Im et al. 2007); curvilinear (Im et al. 2007); like a granddaughter aneurysm (Nagai et al. 2008) and as nebulous area enhancement (Holodny et al. 2003). Extravasated media will accumulate whether the ruptured portion is located in the subarachnoid space, hematoma cavity, or a ventricle. Sometimes the appearance of the extravasated media can be difficult to distinguish from vascular anomalies. However, the presence of an aneurismal teat and accumulated contrast medium within a pre-existing hematoma, together with the absence of a distal connection to other vascular structures, should effectively exclude a vascular malformation (Gosselin & Vieco 1997). Diagnostic delay leads to surgical delay, resulting in progressive brain injury and further risk of re-rupture.

### Comparison with extravasation in DSA

DSA performed within 3 hours of the initial insult carries a high risk of aneurismal re-rupture: up to 23.9% (Kusumi et al. 2005). During DSA, the distal intracarotid pressure rises substantially during injection of contrast medium (Saitoh et al. 1996). Although CTA is considered to be less invasive and to be associated with a lower risk of re-rupture (Tanno et al. 2007), all iodinated media have vasomotor effects after intra-arterial and intra-venous administration: a direct vascular effect attributable to hyperosmolarity; as a consequence of stimulation of the release of endogenous vasomotor mediators and osmolarity-independent direct effects on vascular smooth muscle cells (Limbruno & De Caterina 2003). The possibility that CTA might also be a trigger of rebleeding should not be disregarded, particularly for poor grade SAH in the hyperacute phase.

### Initial management and BP control

Systolic BP in excess of 160 mmHg is reported to be a potential risk factor for rebleeding (Ohkuma et al. 2001). Although the ideal target BP to reduce the risk of rebleeding has not been established, a decrease in systolic BP to <160 mmHg is recommended as reasonable in the latest guidelines (Class IIa; Level of Evidence C) (Connolly et al. 2012). This may be achieved by using a readily titratable drug to balance the risk of stroke, hypertension-related rebleeding and maintenance of cerebral perfusion pressure (Class I; Level of Evidence B) (Connolly et al. 2012).

To mitigate the risk of re-rupture, the importance of guided initial resuscitation should be emphasized as the essential first step of SAH management. Once re-rupture has occurred, the outcome is devastating. At the moment of rupture, intracranial pressure (ICP) abruptly rises and reduces the pressure gradient across the aneurysm wall (Nornes 1973). This phenomenon facilitates hemostasis but continuous bleeding results in intracranial circulatory arrest causing serious ischemic brain injury. Hemostasis ought to be the highest priority for patients in whom there is evidence of ongoing bleeding. In the cases of extravasation reported herein, however, the initial management before and during CTA was not always well documented. One approach to treatment after rebleeding has been identified is to lower systolic BP by 30 mmHg (with a minimum of 140 mmHg), by administering 100–200 ml mannitol and infusing nicardipine (Tsuang et al. 2012).

In the acute phase of poor grade SAH, we mainly used analgesic and anesthetic agents to achieve deep sedation and BP control, and a non-depolarizing muscle relaxant to prevent intracranial hypertension during tracheal intubation. In addition, propofol and nicardipine were used to reduce systolic BP to below 120 mmHg when necessary. This approach was successful in two patients who presented with an initial systolic BP far in excess of 200 mmHg, who subsequently made good recoveries without longstanding consequences of cerebral ischemia. Lowering BP by titrating neuroprotective drugs appeared to be safe and avoided the risk of impairing cerebral perfusion in the acutely injured brain.

### Treatment strategy

The literature suggests that more than half of patients with contrast medium extravasation on CTA did not undergo aneurysm repair and subsequently died. Table 3 shows a summary of other reported cases with ours included (Tsuang et al. 2012; Suzuki et al. 2012; Nakatsuka et al. 2002; Gosselin & Vieco 1997; Nakada et al. 2000; Holodny et al. 2003; Josephson et al. 2004; Ryu et al. 2005; Pérez-Núñez et al. 2006; Hashiguchi et al. 2007; Im et al. 2007; Nagai et al. 2008; Desai et al. 2009; Sholtes et al. 2011). Tsuang et al. concluded that emergency decompressive craniectomy and aneurysm clipping could benefit patients with good neurological status at presentation, and that the outcome for patients with poor grade SAH was bleak irrespective of the management approach (Tsuang et al. 2012). Though rapid intervention is plausible, it takes nearly an hour before surgical decompression can be undertaken after having performed a CTA. Continuous bleeding causes global cerebral ischemia and irreversible brain injury (Komotar et al. 2009; Nornes 1973). Moreover, under these circumstances surgical intervention would carry substantial risk and be technically very challenging, not least because of the difficulties of obtaining as satisfactory surgical field in the face of active hemorrhage and maintaining cardiovascular stability. More intensive resuscitation and strategies to achieve hemostasis and brain protection are warranted before surgery. This is reflected by our efforts to secure the aneurysm for eight potentially salvageable patients: aneurysm repair was achieved in seven, and with appropriate and timely neurocritical care (including induced hypothermia (Kobata et al. 2004)), two made meaningful recoveries.

**Table 3:** **Summary of 44 reported cases of subarachnoid hemorrhage with extravasation of contrast medium seen on computed tomography angiography (CTA), including those reported herein**

Patient characteristics		Valuables
Age	mean ± SD (range)	63.5 ± 15.0 (35–96)
Sex	M/F	16/28
Location	ACoA	16
	ACA	3
	ICA	10
	MCA	9
	BA/VA	4
	PCA	2
Time from onset to CTA	≤1 h	18
	1 h<, ≤2 h	8
	2 h <, ≤3 h	7
	3 h <, ≤6 h	1
	6 h <	3
	NR	7
WFNS Grade before CTA	I	2
	II	6
	III	0
	IV	4
	V	28
	NR	4
Treatment	clipping	15
	coil embolization	3
	no aneurysm secure	26
Outcome	favorable	8
	vegetative state	3
	death	32
	NR	1

The vast majority of patients were of poor grade. Less than half aneurysms were secured and these were associated with poor clinical outcome: 18% survived with a favorable functional status and 72% died.

*WFNS* World Federation of Neurosurgical Societies, *AcoA* anterior communicating artery, *ACA* anterior cerebral artery, *ICA* internal carotid artery, *MCA* middle cerebral artery, *BA* basilar artery, *VA* vertebral artery, *PCA* posterior cerebral artery, *NR* not reported.

### Study limitations

Although potential episodes of re-rupture were carefully examined, the true incidence is difficult to determine. The moment of ictus is often not witnessed and the exact time of onset may not be identified. Sudden deterioration of consciousness and onset of convulsion suggest re-rupture, but these could be overlooked. Neurological deterioration may not be detected in patients who remained comatose after ictus. In addition, 3D-CTA may not be as sensitive a means of detecting extravasation as 4D-CTA (Suzuki et al. 2012). Thus, the incidence of re-rupture may have been underestimated. Multiple rebleeds in the hyperacute phase of SAH are likely to occur more frequently than previously recognized. Patients in “postictal” coma, a state of diminished consciousness soon after aneurysm rupture, may have a good chance of recovery within the first few hours, but they are at high risk of re-rupture. Predicting long-term outcome of poor-grade SAH is difficult in the hyperacute phase. Therefore an intensive supportive approach such as ours is a reasonable means of treating patients with aneurysmal re-rupture to rescue any salvageable patients.

## Conclusions

Continuous or intermittent bleeding from a ruptured intracerebral aneurysm appears to occur more frequently in the hyperacute phase of SAH. Despite an intensive management strategy, we detected active contrast extravasation in 4.5% of patients with hyperacute SAH using 3D–CTA. Generally, the outlook for these patients is bleak, but favorable outcomes can be achieved by immediately securing the aneurysm at decompressive craniectomy, and with timely and intensive neurocritical care.

## Authors’ original submitted files for images

Below are the links to the authors’ original submitted files for images. Authors’ original file for figure 1 Authors’ original file for figure 2 Authors’ original file for figure 3 Authors’ original file for figure 4
