# Supplementary figures and images for: Intracranial extravasation of contrast medium during diagnostic CT angiography in the initial evaluation of subarachnoid hemorrhage: report of 16 cases and review of the literature
Source: Springerplus. 2013 Aug 28;2(1):413. doi: 10.1186/2193-1801-2-413 (PMC3765598; doi:10.1186/2193-1801-2-413)

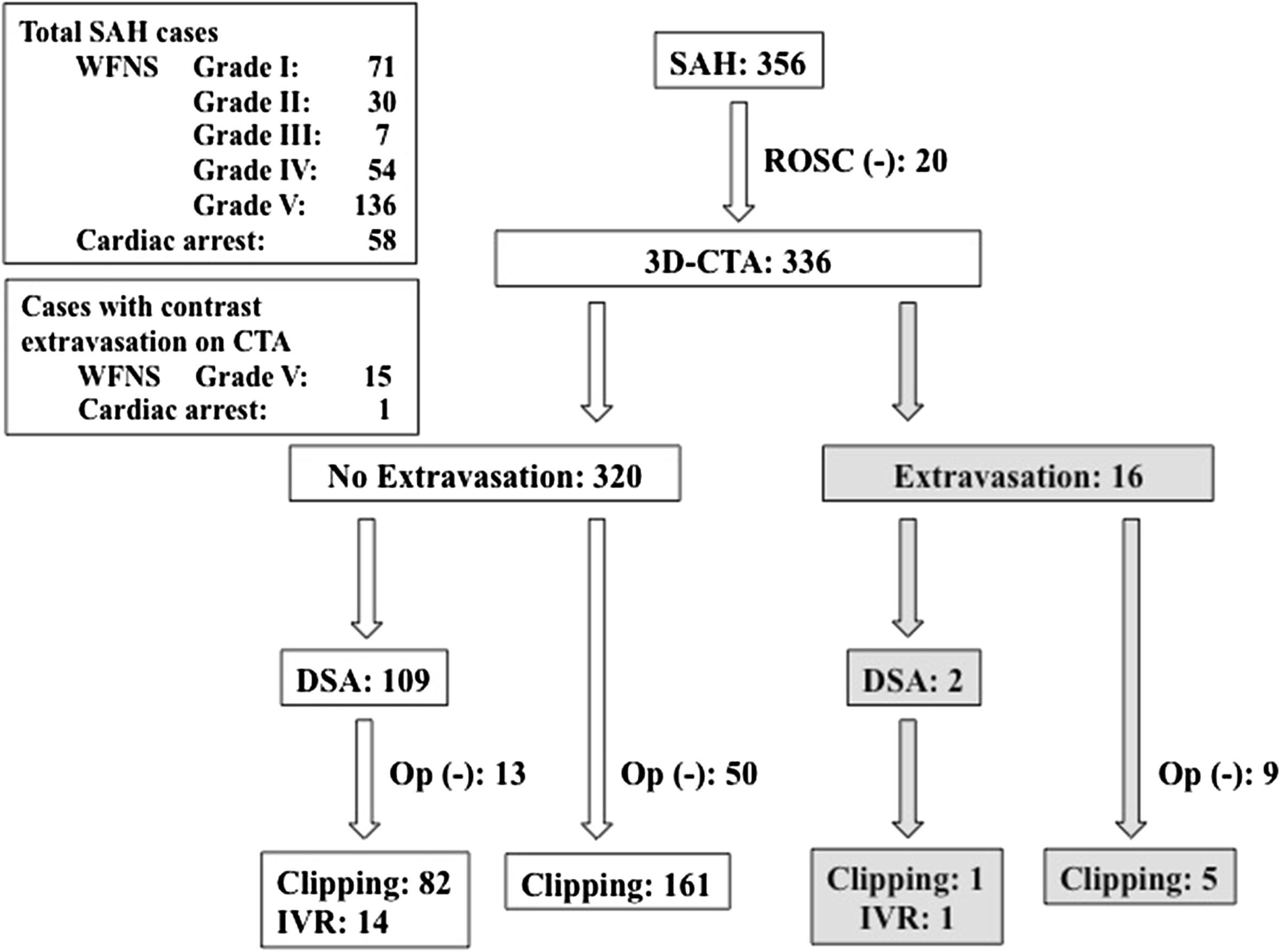

Supplement: Supplementary file 1 — Authors’ original file for figure 1 [file 40064_2013_481_MOESM1_ESM.tif]

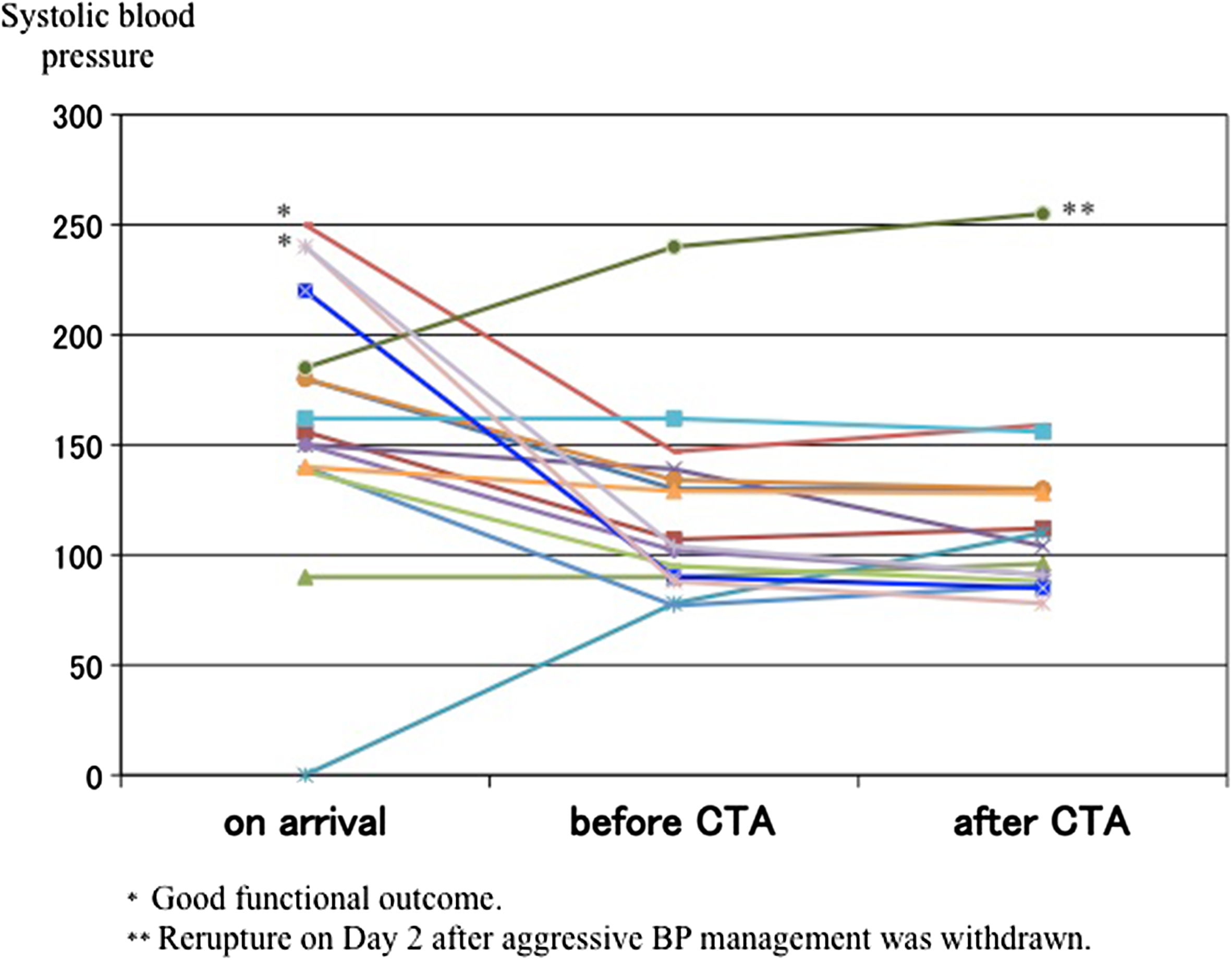

Supplement: Supplementary file 2 — Authors’ original file for figure 2 [file 40064_2013_481_MOESM2_ESM.tif]

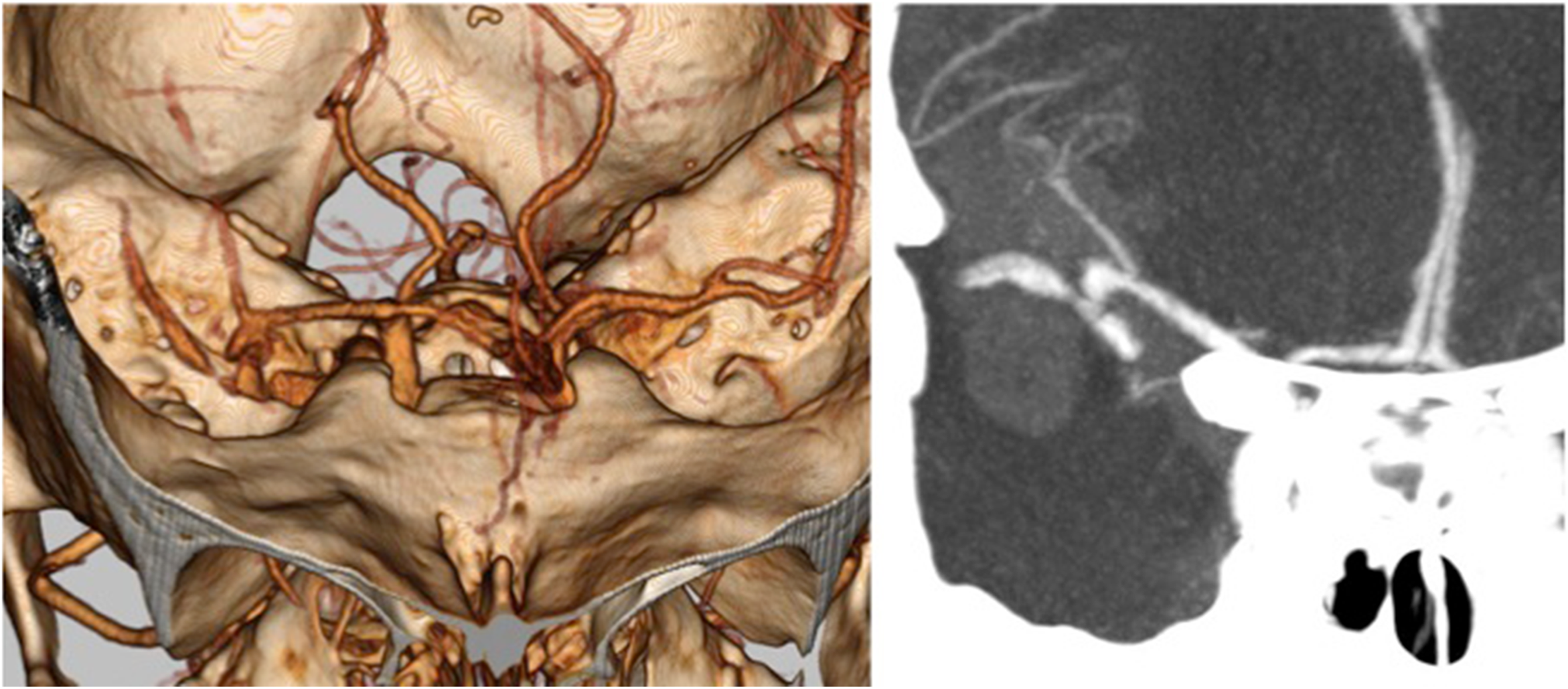

Supplement: Supplementary file 3 — Authors’ original file for figure 3 [file 40064_2013_481_MOESM3_ESM.tif]

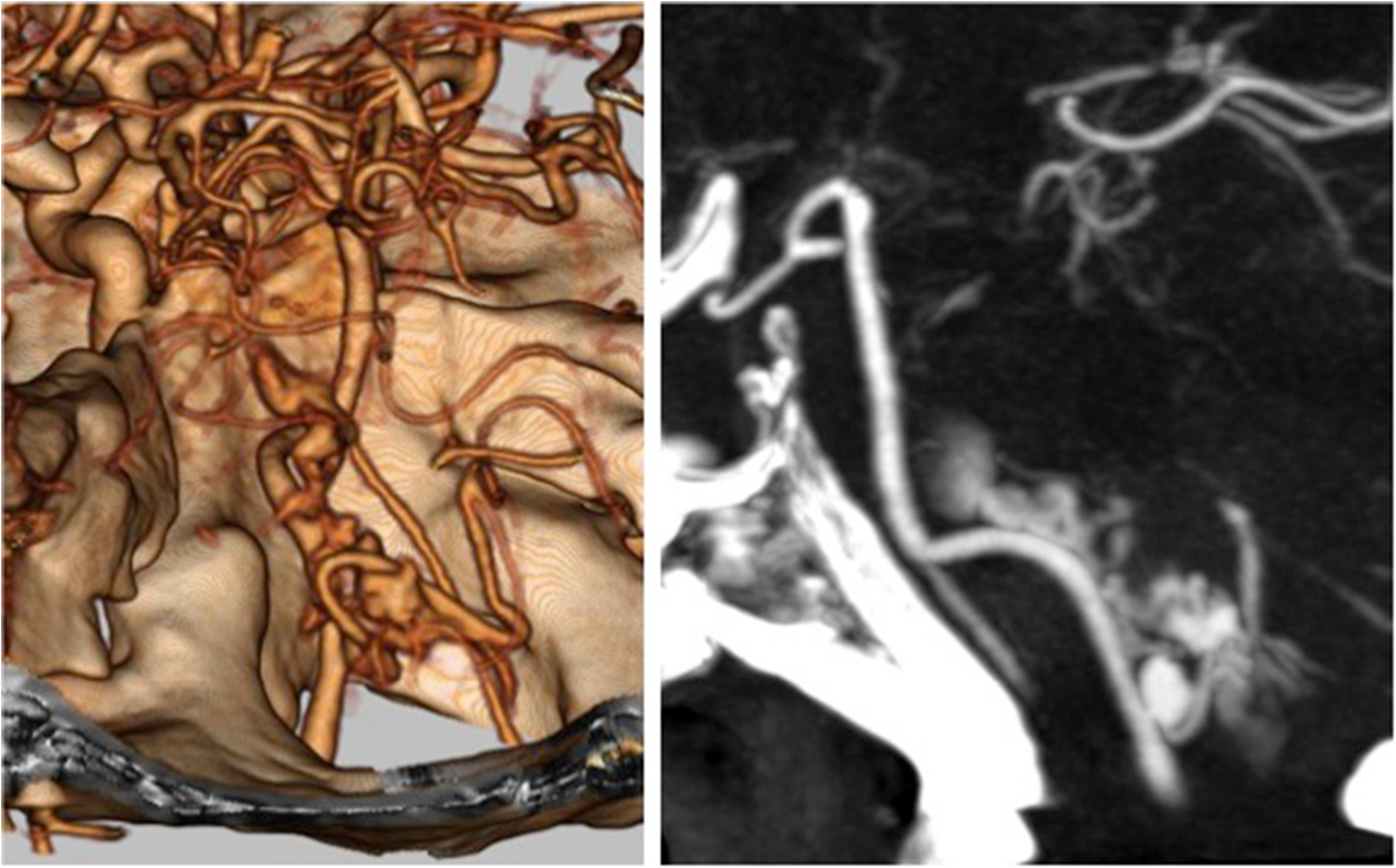

Supplement: Supplementary file 4 — Authors’ original file for figure 4 [file 40064_2013_481_MOESM4_ESM.tif]
